# Viewpoint planning with transition management for active object recognition

**DOI:** 10.3389/fnbot.2023.1093132

**Published:** 2023-02-24

**Authors:** Haibo Sun, Feng Zhu, Yangyang Li, Pengfei Zhao, Yanzi Kong, Jianyu Wang, Yingcai Wan, Shuangfei Fu

**Affiliations:** ^1^Faculty of Robot Science and Engineering, Northeastern University, Shenyang, China; ^2^Key Laboratory of Opto-Electronic Information Processing, Chinese Academy of Sciences, Shenyang, China; ^3^Shenyang Institute of Automation, Chinese Academy of Sciences, Shenyang, China; ^4^Institutes for Robotics and Intelligent Manufacturing, Chinese Academy of Sciences, Shenyang, China; ^5^University of Chinese Academy of Sciences, Beijing, China

**Keywords:** active object recognition, viewpoint planning, deterministic policy gradient, twin delayed deep deterministic policy gradient, viewpoint transition management, reinforcement learning

## Abstract

Active object recognition (AOR) provides a paradigm where an agent can capture additional evidence by purposefully changing its viewpoint to improve the quality of recognition. One of the most concerned problems in AOR is viewpoint planning (VP) which refers to developing a policy to determine the next viewpoints of the agent. A research trend is to solve the VP problem with reinforcement learning, namely to use the viewpoint transitions explored by the agent to train the VP policy. However, most research discards the trained transitions, which may lead to an inefficient use of the explored transitions. To solve this challenge, we present a novel VP method with transition management based on reinforcement learning, which can reuse the explored viewpoint transitions. To be specific, a learning framework of the VP policy is first established *via* the deterministic policy gradient theory, which provides an opportunity to reuse the explored transitions. Then, we design a scheme of viewpoint transition management that can store the explored transitions and decide which transitions are used for the policy learning. Finally, within the framework, we develop an algorithm based on twin delayed deep deterministic policy gradient and the designed scheme to train the VP policy. Experiments on the public and challenging dataset GERMS show the effectiveness of our method in comparison with several competing approaches.

## 1. Introduction

**Figure 1:**
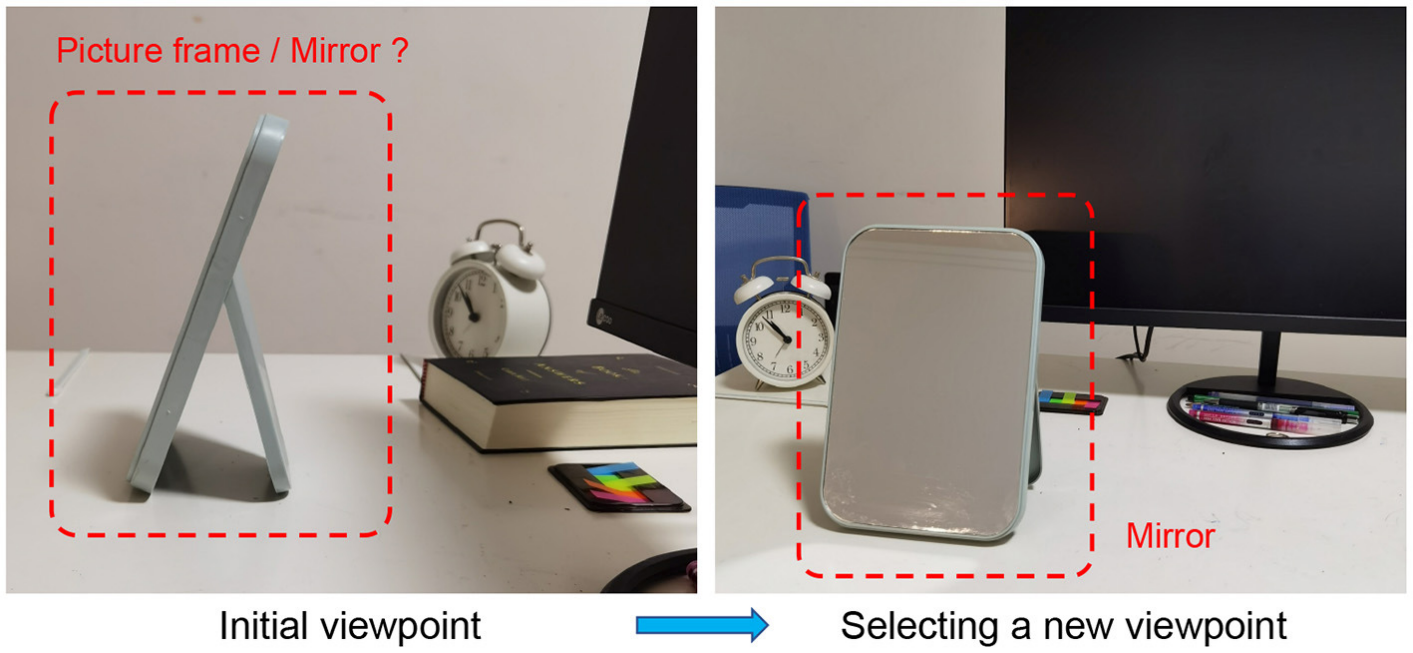
An example illustrating the active preception process of people.

Visual object recognition has a wide range of applications e.g., automatic driving (Behl et al., 2017), robotics (Stria and Hlavác, 2018), medical diagnostic (Duan et al., 2019), environmental perception (Roynard et al., 2018), etc. Most recognition systems merely take a single viewpoint image as input and produce a category label estimate as output (Jayaraman and Grauman, 2019). It is prone to the recognition errors when the image can not provide sufficient information. In contrast, the visual behavior of people is an active process so as to more clearly perceive their surroundings. As shown in Figure 1, in daily life, people can intelligently observe an object from different viewpoints to determine the identity of the object. Similarly, if the viewpoint of an agent can be adjusted (e.g., mobile robots and autonomous vehicles), more valuable information will be obtained to boost the recognition performance.

As a branch of active vision (Parr et al., 2021), active object recognition (AOR) (Patten et al., 2015; Wu et al., 2015; Potthast et al., 2016; Van de Maele et al., 2022) is a typical technology to realize the above idea, which aims to collect additional clues by purposefully changing the viewpoint of an agent to improve the quality of recognition. Andreopoulos and Tsotsos (2013) and Zeng et al. (2020) review a series of classical AOR methods. One of the most concerned problems in AOR is viewpoint planning (VP) that refers to developing a policy to determine the next viewpoints of the agent. In recent years, researchers mainly focus on using reinforcement learning to solve the VP problem (Becerra et al., 2014; Malmir et al., 2015; Malmir and Cottrell, 2017; Liu et al., 2018a), namely to use the viewpoint transitions explored by the agent to train the VP policy. Becerra et al. (2014) formally define object recognition as a partially observable Markov decision process problem and uses stochastic dynamic programming to address the problem. As a pioneering work, Malmir et al. (2015) provide a public AOR dataset called GERMS that includes 136 objects with different view images and develops a deep Q-learning (DQL) system to learn to actively verify objects by using standard back-propagation and Q-learning. In the same way, Liu et al. (2018a) design a hierarchical local-receptive-field architecture to predict object label and learns a VP policy by combining extreme learning machine and Q-learning. Similar to Becerra et al. (2014), AOR is also modeled as a partially observable Markov decision process by Malmir and Cottrell (2017). The difference is that a belief tree search is built to find near-optimal action values which correspond to the next best viewpoints. These VP methods explore discrete viewpoint space, which may introduce significant quantization errors. Hence, Liu et al. (2018b) present a continuous VP method based on trust region policy optimization (TRPO) (Schulman et al., 2015) and adopts extreme learning machine (Huang et al., 2006) to reduce computational complexity. It shows a promising result on the GERMS dataset compared to the discrete VP methods. However, due to the on-policy characteristic of TRPO, the trained viewpoint transitions will be discarded by the agent, which may lead to an inefficient use of the explored transitions.

The deterministic policy gradient theory (Silver et al., 2014) is proposed for reinforcement learning with continuous actions and introduces an off-policy actor-critic algorithm (OPDAC-Q) to learn a deterministic target policy. Lillicrap et al. (2015) present a deep deterministic policy gradient (DDPG) approach that combines deterministic policy gradient with DQN (Mnih et al., 2013, 2015) to learn policies in high-dimensional continuous action spaces. Fujimoto et al. (2018) contribute a mechanism that takes the minimum value between a pair of critics in the actor-critic algorithm of Silver et al. (2014) to tackle the function approximation errors. The deterministic policy gradient theory has been widely applied in various fields, such as electricity market (Liang et al., 2020), vehicle speed tracking control (Hao et al., 2021), fuzzy PID controller (Shi et al., 2020), quadrotor control (Wang et al., 2020), energy efficiency (Zhang et al., 2020), and autonomous underwater vehicles (Sun et al., 2020; Wu et al., 2022). However, to our best knowledge, it has never been employed in the AOR task.

In this work, we present a novel continuous VP method with transition management based on reinforcement learning. This method can efficiently use the explored viewpoint transitions to learn the continuous VP policy. Concretely, a learning framework of the continuous VP policy is established using the deterministic policy gradient theory, which provides an opportunity to reuse the explored transitions owing to the off-policy characteristic of the theory. Then, we design a scheme of viewpoint transition management that can store the explored transitions and decide which transitions are used for the policy learning. The scheme is implemented by introducing and improving the prioritized experience replay technology (Schaul et al., 2016). The improvements include: (1) We improve the estimation approach of temporal difference (TD) error with the clipped double Q-learning algorithm (Fujimoto et al., 2018) so as to adapt to our continuous VP framework. (2) We utilize importance-sampling to correct the estimation bias of TD error produced by the prioritized replay. Finally, within the framework, we develop an algorithm based on twin delayed deep deterministic policy gradient (TD3) (Fujimoto et al., 2018) and the designed scheme to train the continuous VP policy. Experimental results on the public dataset GERMS demonstrate the effectiveness of the proposed VP method.

The key contributions of this work are

- A novel continuous VP method with transition management for AOR is presented to solve the problem of inefficient use of the explored viewpoint transitions in the existing continuous VP method.
- We establish a learning framework of the continuous VP policy *via* the deterministic policy gradient theory.
- A scheme of viewpoint transition management is designed, which is implemented by introducing and improving the prioritized experience replay technology.
- We develop an algorithm based on twin delayed deep deterministic policy gradient and the designed scheme to train the continuous VP policy.

The rest of this paper is structured as follows: Section 2 formulates the VP problem. Section 3 details the proposed framework for the solution of the problem. Finally, the implementation and experimental results, as well as conclusions are further provided in Sections 4, 5.

## 2. Problem definition

An AOR system mounted on an automatic mobile agent allows the agent to identify an object by dealing with the images captured from different viewpoints. Suppose at the initial time *t* = 0, an object to be identified is given from an object library containing *M* objects and the agent captures an image *I*_Φ_0__ from the initial viewpoint Φ_0_. The classifier C(·) in the AOR system will give a probability prediction $C\left(I_{\Phi_{0}}\right)$ of the object according to the image *I*_Φ_0__. $C\left(I_{\Phi_{0}}\right)$ is a *M* dimensional vector where every element denotes recognition probability of different objects in the library. When the prediction is uncertain [i.e., the maximum probability in $C\left(I_{\Phi_{0}}\right)$ is less than the preset threshold], the agent will move to explore more viewpoints to improve recognition performance. This requires the system plans a relative movement action *a*_*t*_ for the agent to obtain a new viewpoint Φ_*t*+1_ = Φ_*t*_+*a*_*t*_. The new image *I*_Φ_*t*+1__ captured from the viewpoint Φ_*t*+1_ will be used for the recognition again. This process is repeated several times until a stop condition (e.g., planning up to *T*_*max*_ time steps or reaching the preset probability threshold) is reached.

An undesirable planning action may make it difficult for the agent to capture useful images for recognition. Therefore, we need to find an effective VP policy for the AOR system. For this purpose, the VP problem is considered as a reinforcement learning paradigm which can be formulated as a Markov decision process. The process is described with a six-element tuple <S,A,r,P,γ,u>.

- *S* represents a set of continuous states in which each state *s* is produced by the predictions of corresponding images captured from different viewpoints.
- *A* is a set of continuous actions which are determined by the agent. Each action *a* in the set is used for the agent to get a new viewpoint.
- *r*:*S*×*A* → ℝ is a reward function designed to evaluate the quality of selecting a viewpoint.
- P:S×A×S→[0,1] denotes the transition probability. It describes the possibility of transferring to the subsequent state *s*, after the action *a* is selected in the state *s*.
- γ ∈ [0, 1] is a discount factor used to adjust the attention between present and future rewards.
- *u*:*S*→*A* is a deterministic continuous VP policy [i.e., *a* = *u*(*s*)] that can generate an action for the agent to get a new viewpoint in a certain state.

The VP problem is transformed to solve the optimal policy u^*^ in the setting of reinforcement learning.

## 3. Method

### 3.1. Overview

In reinforcement learning, the optimal policy u^*^ can be achieved by maximizing the expected return over all episodes. At any time step *t* of each episode, with a given state *s*_*t*_∈*S*, the agent plans an action *a*_*t*_∈*A* according to its current policy *u* (*a*_*t*_ = *u*(*s*_*t*_)), receiving a reward *r*(*s*_*t*_, *a*_*t*_) and the new state $s_{t+1}\sim P\left(s_{t+1}|s_{t},a_{t}\right)$. ((*s*_*t*_, *a*_*t*_, *r*_*t*_, *s*_*t*+1_) is called the viewpoint transition in the AOR task.) The return is defined as the cumulative discounted reward $\sum_{i=t}^{T}\gamma^{i-t}r\left(s_{i},a_{i}\right)$ where *T* is the end time step of planning. Let $Q^{u}\left(s_{t},a_{t}\right)$ be the expected return when performing action *a*_*t*_ in state *s*_*t*_ under the policy *u*. $Q^{u}\left(s_{t},a_{t}\right)$ is defined as


(1) $$Q^{u}(s_{t},a_{t})=\underset{s_{t+1}\sim P(s_{t+1}|s_{t},a_{t})}{E}[\sum_{i=t}^{T}\gamma^{i-t}r(s_{i},a_{i})|s_{t},a_{t}]$$

which is known as the action value function. u^*^ can be solved by maximizing the expected value of Equation (1) over the whole state space


(2) $$u^{*}=\underset{u}{\max}E_{s_{t}\sim d(\cdot )}[Q^{u}(s_{t},a_{t})|a_{t}=u(s_{t})]$$

where *d*(·) is the state probability density of Markov decision process in steady state distribution (Bellemare et al., 2017).

We assume the deterministic continuous VP policy *u* is parameterized by θ and denote it as *u*(*s*; θ). Naturally, Equation (2) can be transformed to an optimization with respect to θ that maximize the objective


(3) $$\begin{array}{l} J\left(\theta \right)={\text{𝔼}}_{s_{t}\sim d\left(\cdot \right)}\left[Q^{u}\left(s_{t},a_{t}\right)|a_{t}=u\left(s_{t};\theta \right)\right]. \end{array}$$

To solve the optimization of Equation (3), the deterministic policy gradient theory (Silver et al., 2014) is introduced to iteratively update the parameters θ by taking the gradient of Equation (3)


(4) $$\begin{array}{l} {▿}_{\theta }J\left(\theta \right)={\text{𝔼}}_{s_{t}\sim d\left(\cdot \right)}\left[{▿}_{\theta }u\left(s_{t};\theta \right){▿}_{a}Q^{u}\left(s_{t},a_{t}\right)|a_{t}=u\left(s_{t};\theta \right)\right]. \end{array}$$

**Figure 2:**
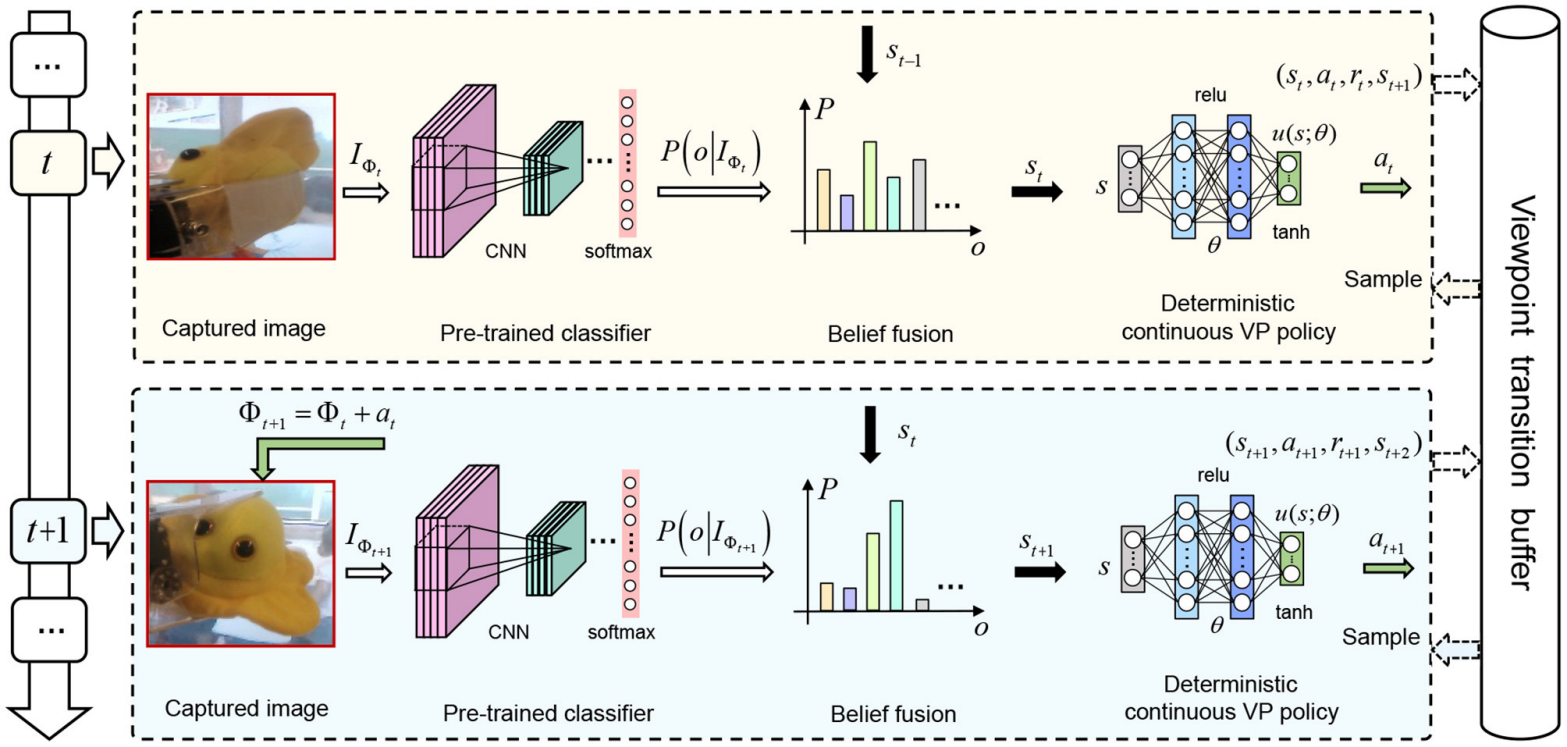
The pipeline of active object recognition based on deterministic continuous viewpoint planning. The deterministic policy gradient theory (Silver et al., 2014) is introduced to build a framework of continuous viewpoint planning. We design a scheme of viewpoint transition management to store and replay the explored viewpoint transitions. Within the framework, we develop an algorithm based on TD3 (Fujimoto et al., 2018) and the scheme to train the VP policy network. During the training, the agent stores the explored viewpoint transition (*s*_*t*_, *a*_*t*_, *r*_*t*_, *s*_*t*+1_) in the viewpoint transition buffer and samples a mini-batch transitions from it to train the VP policy network at each time step.

We utilize (Equation 4) as a framework to learn the optimal deterministic continuous VP policy $u\left(s_{t};\theta^{*}\right)$ for AOR. The reason why this framework can reuse the explored viewpoint transitions is the off-policy characteristic of the deterministic policy gradient theory, i.e., the viewpoint transitions explored by any policy can be used for the calculation of the gradient in Equation (4), because the gradient is only related to the distribution of state *s*_*t*_ (Silver et al., 2014). The pipeline of our AOR is shown in Figure 2 where the VP policy *u*(*s*_*t*_; θ) is represented by a three-layer fully-connected neural network with the parameters θ. The policy network *u*(*s*_*t*_; θ) takes a state *s*_*t*_ as input and outputs a deterministic action *a*_*t*_ = *u*(*s*_*t*_; θ). In the following, the representations of state *s*_*t*_ and reward function *r*(*s*_*t*_, *a*_*t*_) will be elaborated. Additionally, we will design a scheme of viewpoint transition management and develop a training algorithm based on twin delayed deep deterministic policy gradient (TD3) (Fujimoto et al., 2018) and the scheme for the learning of $u\left(s_{t};\theta^{*}\right)$ within the framework.

### 3.2. Recognition state

As shown in Figure 2, we first use a convolutional neural network (CNN) model to extract features from the captured image *I*_Φ_*t*__ and then recognize the concerned objects with a *softmax* layer added the top of the CNN model. The CNN model and the *softmax* layer constitute a classifier C(·) which is pre-trained with the images from different viewpoints of the concerned objects. The parameters of the classifier are fixed when training the VP policy network. The classifier outputs a belief vector $C\left(I_{\Phi_{t}}\right)$ where every element denotes recognition probability of different objects. The *oth* element in the vector is represented as *P*(*o*|*I*_Φ_*t*__) where *o* = 1, 2, ..., *M* is the object label. The recognition state *s*_*t*_ is a posterior probability distribution over different objects at time step *t*, which is produced by the captured images. It is also expressed as a vector where the *oth* element is *P*(*o*|*I*_Φ_0__, *I*_Φ_1__, ..., *I*_Φ_*t*__), *o* = 1, 2, ..., *M*. According to naive Bayes (Paletta and Pinz, 2000), *P*(*o*|*I*_Φ_0__, *I*_Φ_1__, ..., *I*_Φ_*t*__) is given as


(5) $$\begin{array}{l} \xi_{t}P\left(o|I_{\Phi_{t}}\right)P\left(o|I_{\Phi_{0}},I_{\Phi_{1}},...,I_{\Phi_{t-1}}\right) \end{array}$$

where ξ_*t*_ is a normalizing coefficient.

### 3.3. Reward function

Reward function *r*(*s*_*t*_, *a*_*t*_) (denoted as *r*_*t*_ for simplicity) is used to evaluate the quality of selecting a viewpoint. As described in Section 3.2, state is a posterior probability distribution over different objects. The flatter the distribution is, the stronger the recognition uncertainty is. To quantify the uncertainty, information entropy (Zhao et al., 2016; Liu et al., 2018b) is utilized and the uncertainty in state *s*_*t*_ is denoted as $H\left(s_{t}\right)=-\underset{o}{\sum }P\left(o|I_{\Phi_{0}},I_{\Phi_{1}},...,I_{\Phi_{t}}\right)\log P\left(o|I_{\Phi_{0}},I_{\Phi_{1}},...,I_{\Phi_{t}}\right)$. The purpose of AOR is to reduce the uncertainty of recognition through viewpoint planning. Therefore, we can design the reward function according to the change of uncertainty before and after viewpoint selection. The resulting reward function is


(6) $$\begin{array}{l} r_{t}=\{\begin{array}{cc} -1, & {ô}_{t+1}\neq o^{*}\\ 0, & {ô}_{t+1}=o^{*},H\left(s_{t+1}\right)\geq H\left(s_{t}\right)\\ 1, & {ô}_{t+1}=o^{*},H\left(s_{t+1}\right)<H\left(s_{t}\right) \end{array} \end{array}$$

where o^*^ is the object label and ô_*t*+1_ = *argmax*_*o*_*P*(*o*|*I*_Φ_0__, *I*_Φ_1__, ..., *I*_Φ_*t*+1__) is the predicted result. When the predicted result is right (${ô}_{t+1}=o^{*}$) and the uncertainty is reduced (*H*(*s*_*t*+1_) < *H*(*s*_*t*_)), it indicates that this viewpoint selection is valuable for recognition. On the contrary, other situations mean that this viewpoint selection is not good.

### 3.4. Viewpoint transition management

The agent can obtain a transition (*s*_*t*_, *a*_*t*_, *r*_*t*_, *s*_*t*+1_) after a viewpoint selection and use it for the learning of the continuous VP policy. In the TRPO-based VP method (Liu et al., 2018b), the obtained viewpoint transitions will be discarded after they are trained due to the on-policy characteristic of TRPO. It leads to a low efficient use of the obtained transitions. In our work, the deterministic policy gradient theory (Silver et al., 2014) allows the agent to reuse the obtained transitions. Therefore, to make full use of the obtained viewpoint transitions, the experience replay (ER) (Lin, 1992; Schaul et al., 2016) technology is adopted and improved to implement a scheme of viewpoint transition management. The scheme includes viewpoint transition storage and viewpoint transition reuse.

#### 3.4.1. Viewpoint transition storage

To store the obtained viewpoint transitions, we build a viewpoint transition buffer with a capacity of *K* in the light of Lin (1992) and Schaul et al. (2016). *K* is generally within 10^4^~10^6^. Once the buffer is full of transitions, the old ones will be replaced by the newly generated transitions.

#### 3.4.2. Viewpoint transition reuse

The key of viewpoint transition reuse is to decide which transitions to reuse. Lin (1992) adopt a uniform sampling strategy that means the sampling probability of each transition in the buffer is the same. However, those transitions with greater temporal difference (TD) errors are obviously more surprising to the agent and should be sampled with a higher probability (Schaul et al., 2016). Hence, Schaul et al. (2016) present a prioritized experience replay (PER) technology that can quantify the surprising level (priority) of each transition by the TD error and convert the priority into the corresponding sampling probability. Here, we employ the PER technology to sample the viewpoint transitions in the buffer. Concretely, the probability of sampling the *i*th stored viewpoint transition is given as


(7) $$\begin{array}{l} P\left(i\right)=\frac{p_{i}^{\lambda }}{\overset{K}{\underset{l=1}{\sum }}p_{l}^{\lambda }} \end{array}$$

where $p_{i}^{\lambda }>0$ is the priority of the *i*th transition. The exponent λ indicates how much prioritization is used, with λ = 0 corresponding to the uniform case. Proportional prioritization is defined with


(8) $$\begin{array}{l} p_{i}=|{\hat{\delta }}_{i}|+\epsilon \end{array}$$

where ${\hat{\delta }}_{i}$ is the TD error of the *i*th transition and ϵ is a small positive value that prevents transitions with error of 0 from not being sampled. The estimation of TD error in PER is based on the double DQN algorithm (Mnih et al., 2015).


(9) $$\begin{array}{l} {\hat{\delta }}_{i}=r_{t}^{\left(i\right)}+\gamma Q\left(s_{t+1}^{\left(i\right)},argmax_{a}Q\left(s_{t+1}^{\left(i\right)},a;\omega \right);\omega^{-}\right)-Q\left(s_{t}^{\left(i\right)},a_{t}^{\left(i\right)};\omega \right) \end{array}$$

where *Q*(*s*_*t*_, *a*_*t*_; ω) and $Q\left(s_{t},a_{t};\omega^{-}\right)$ are value function network and target value function network respectively. However, it is only applicable to discrete viewpoint planning, not to our continuous case. Inspired by Fujimoto et al. (2018), we improve the estimation method of TD error with the clipped double Q-learning algorithm so as to adapt to our deterministic continuous VP framework. The improved TD error is


(10) $$\begin{array}{l} {\hat{\delta }}_{i}=|{ŷ}_{t}^{\left(i\right)}-Q\left(s_{t}^{\left(i\right)},a_{t}^{\left(i\right)};\omega_{1}\right)|+|{ŷ}_{t}^{\left(i\right)}-Q\left(s_{t}^{\left(i\right)},a_{t}^{\left(i\right)};\omega_{2}\right)| \end{array}$$

where ${ŷ}_{t}^{\left(i\right)}=r_{t}^{\left(i\right)}+\gamma \min_{j=1,2}Q\left(s_{t+1}^{\left(i\right)},u\left(s_{t+1}^{\left(i\right)};\theta^{-}\right);\omega_{j}^{-}\right)$ is TD target. *Q*(*s*_*t*_, *a*_*t*_; ω_1_) and *Q*(*s*_*t*_, *a*_*t*_; ω_2_) are two value function networks, and $Q\left(s_{t},a_{t};\omega_{1}^{-}\right)$ and $Q\left(s_{t},a_{t};\omega_{2}^{-}\right)$ are their corresponding target value function networks. $u\left(s_{t};\theta^{-}\right)$ is the target policy network. These networks will be elaborated in the next subsection.

In addition, we find that the estimation of TD error is biased due to the prioritized sampling. It is known that Bellman optimality equation (Sutton and Barto, 2018) is $Q\left(s_{t},a_{t}\right)={\text{𝔼}}_{s_{t+1}\sim P\left(s_{t+1}|s_{t},a_{t}\right)}\left[r_{t}+\gamma \underset{a}{\max}Q\left(s_{t+1},a\right)\right]$ where $y_{t}=r_{t}+\gamma \underset{a}{\max}Q\left(s_{t+1},a\right)$ is TD target. Obviously, the distribution $s_{t+1}\sim P\left(s_{t+1}|s_{t},a_{t}\right)$ is changed by using the prioritized sampling, which introduces bias to the estimation of the expected value *Q*(*s*_*t*_, *a*_*t*_). Thus, we correct the bias with importance-sampling weight $\rho =\frac{P}{D}$ where D is the new distribution of *s*_*t*+1_ generated due to the use of prioritized sampling. Then Bellman optimality equation is transformed to $Q\left(s_{t},a_{t}\right)={\text{𝔼}}_{s_{t+1}\sim D\left(s_{t+1}|s_{t},a_{t}\right)}[\rho \left(r_{t}+\gamma \underset{a}{\max}Q\left(s_{t+1},a\right)\right]$ where $\rho (r_{t}+\gamma \underset{a}{\max}Q\left(s_{t+1},a\right)$ is TD target with bias correction denoted as $y_{t}^{corr}$. And TD error is transformed to $\delta =y_{t}^{corr}-Q\left(s_{t},a_{t}\right)$. Similar, in our scheme, the importance-sampling weight of the *ith* viewpoint transition in the buffer is


(11) $$\begin{array}{l} \rho_{i}=\frac{1}{K\cdot P\left(i\right)} \end{array}$$

where *K* is the capacity of the buffer. Our clipped double Q-learning based TD error and TD target are corrected as


(12) $$\begin{array}{l} {\hat{\delta }}_{i}^{corr}=|{\hat{y}}_{t}^{corr(i)}-Q(s_{t}^{\left(i\right)},a_{t}^{\left(i\right)};\omega_{1})|+|{\hat{y}}_{t}^{corr(i)}-Q(s_{t}^{\left(i\right)},a_{t}^{\left(i\right)};\omega_{2})|\\ {\hat{y}}_{t}^{corr(i)}=\rho_{i}(r_{t}^{\left(i\right)}+\gamma \underset{j=1,2}{\min}Q(s_{t+1}^{\left(i\right)},u(s_{t+1}^{\left(i\right)};\theta^{-});\omega_{j}^{-})). \end{array}$$

To avoid expensive sweeps over the entire viewpoint transition buffer, priorities are only updated for the transitions that are sampled according to Schaul et al. (2016). In addition, the new transitions will be put in the buffer with maximal priority in order to guarantee that all transitions are seen at least once.

### 3.5. Training the policy network

In this section, we resort twin delayed deep deterministic policy gradient (TD3) (Fujimoto et al., 2018) and the scheme designed in Section 3.4 to develop a training algorithm for the solution of the optimal VP policy parameters θ^*^. To this end, we use the gradient (Equation 4) to iteratively update θ: θ = θ+α▿_θ_*J*(θ). α is the learning rate. The core task is to solve the gradient ▿_θ_*J*(θ). We therefore employ Monte Carlo method to replace the expected operator in Equation (4) in an approximate manner. Specifically, we sample *N* transitions from the viewpoint transition buffer using Equation (7) to calculate


(13) $$\begin{array}{l} {▿}_{\theta }J\left(\theta \right)\approx \frac{1}{N}\overset{N}{\underset{i=1}{\sum }}\left[\rho_{i}\cdot {▿}_{\theta }u\left(s_{t}^{\left(i\right)};\theta \right){▿}_{a}Q^{u}\left(s_{t}^{\left(i\right)},u\left(s_{t}^{\left(i\right)};\theta \right)\right)\right]. \end{array}$$

According to TD3, we approximately represent the value function $Q^{u}\left(s_{t},a_{t}\right)$ in Equation (13) by a three-layer fully-connected neural network *Q*(*s*_*t*_, *a*_*t*_; ω) with the parameters ω. The network takes the state *s*_*t*_ and the action *a*_*t*_ as input and outputs the function value *Q*(*s*_*t*_, *a*_*t*_; ω). By updating the parameters ω, the value function corresponding to the VP policy *u* can be obtained.

**Figure 3:**
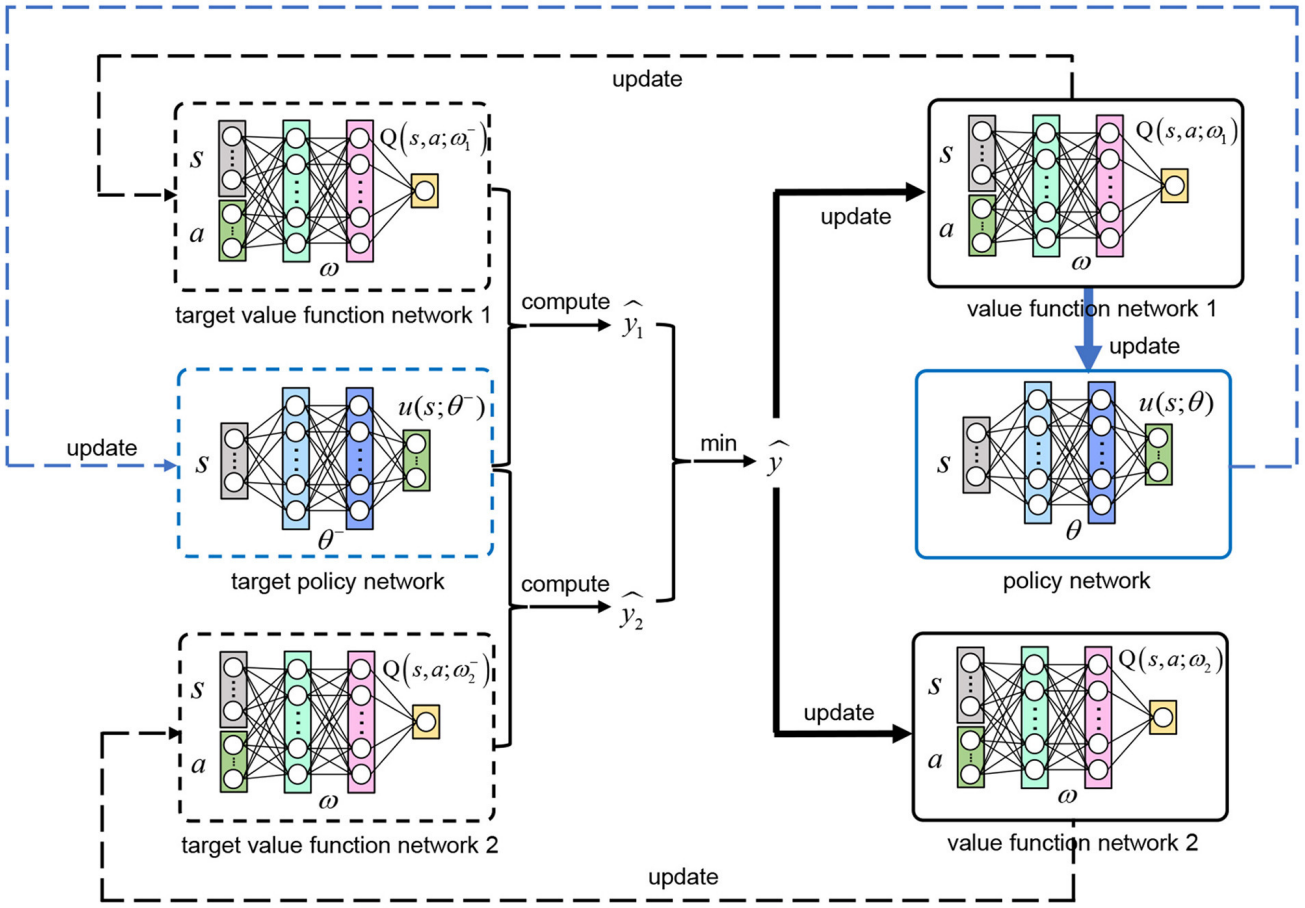
The relationship between the six networks. The TD target ŷ is estimated with the target value function network 1 and 2 using our clipped double Q-learning and bias correction based algorithm (Equation 12), which is used to update the value function network 1 and 2. With the gradient of *Q*(*s*_*t*_, *a*_*t*_; ω_1_) to *a*, the policy network is updated with Equation (13). Three target networks ($u\left(s_{t};\theta^{-}\right),Q\left(s_{t},a_{t};\omega_{1}^{-}\right),Q\left(s_{t},a_{t};\omega_{2}^{-}\right)$) adopt soft updates according to their corresponding evaluation networks (*u*(*s*_*t*_; θ), *Q*(*s*_*t*_, *a*_*t*_; ω_1_), *Q*(*s*_*t*_, *a*_*t*_; ω_2_)).

In order to better train the policy network *u*(*s*_*t*_; θ), we follow TD3 to build six neural networks in total: policy network *u*(*s*_*t*_; θ), value function network 1 *Q*(*s*_*t*_, *a*_*t*_; ω_1_), value function network 2 *Q*(*s*_*t*_, *a*_*t*_; ω_2_) and their corresponding target networks [target policy network $u\left(s_{t};\theta^{-}\right)$, target value function network 1 $Q\left(s_{t},a_{t};\omega_{1}^{-}\right)$, target value function network 2 $Q\left(s_{t},a_{t};\omega_{2}^{-}\right)$]. After the training, the policy network *u*(*s*_*t*_; θ) is the optimal deterministic continuous VP policy we want. The other networks only serve as auxiliary training. Figure 3 shows the relationship between the six networks.

The value function networks can be updated with the aforementioned *N* samples by minimizing the objective


(14) $$\begin{array}{l} L\left(\omega_{j}\right)=\frac{1}{2N}\overset{N}{\underset{i=1}{\sum }}{\left({ŷ}_{t}^{corr\left(i\right)}-Q\left(s_{t}^{\left(i\right)},a_{t}^{\left(i\right)};\omega_{j}\right)\right)}^{2} \end{array}$$

where j is 1 or 2. ${ŷ}_{t}^{corr}$ is the corrected TD target proposed in Equation (12).

Our whole algorithm to train the deterministic continuous VP policy network is summarized in Algorithm 1. Once the optimal parameters θ^*^ are obtained after the training, we can use them for the practical AOR task. Given a state *s*_*t*_, the planned action is $a_{t}^{*}=u\left(s_{t};\theta^{*}\right)$, and the next best viewpoint of the agent is $\Phi_{t+1}=\Phi_{t}+a_{t}^{*}$.

**Algorithm 1:**
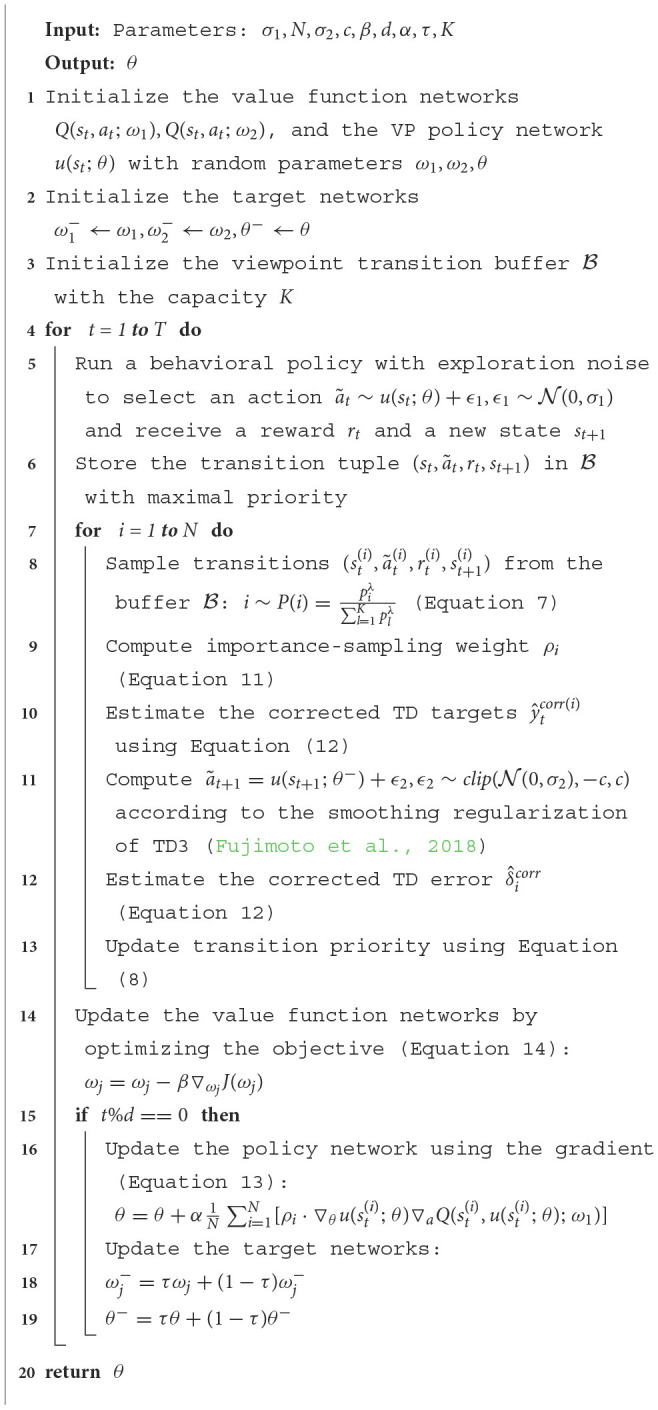
Training the deterministic continuous VP policy network.

## 4. Experiments

This section first provides details about the experimental dataset and implementation, and then reports the experimental results along with some analyzes.

### 4.1. Dataset and metric

**Figure 4:**
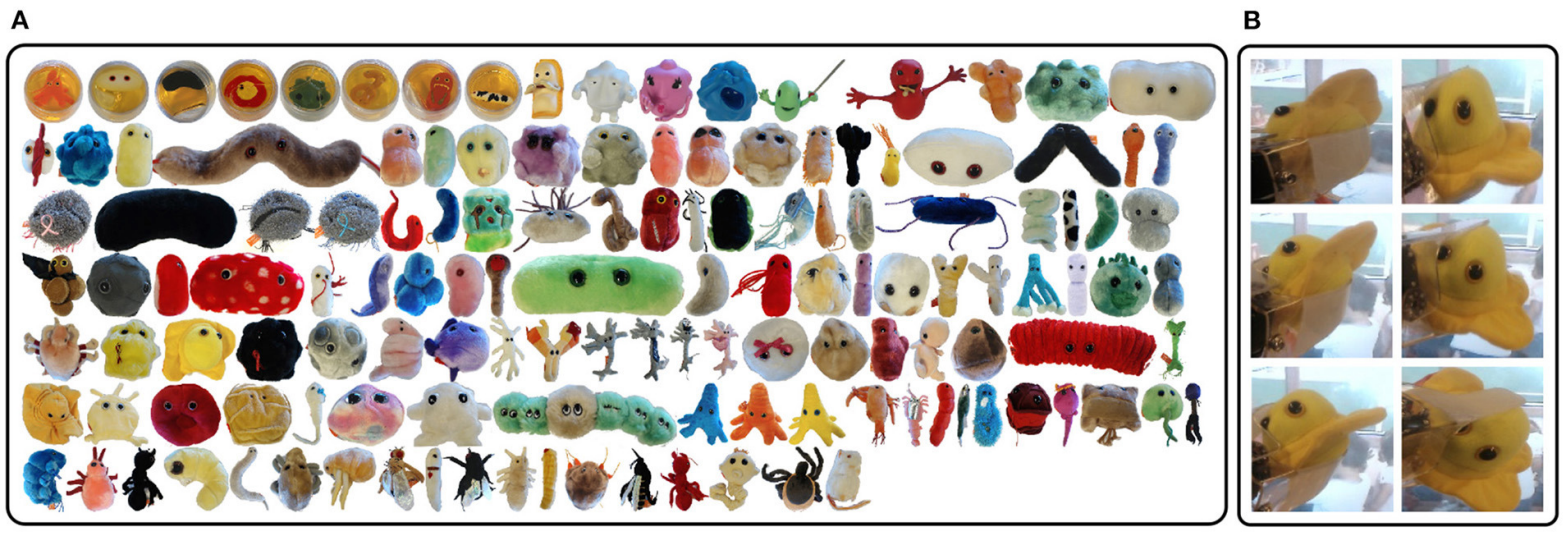
The GERMS dataset. **(A)** One hundred and thirty six object instances. **(B)** Recorded images of different joint positions in each track.

We evaluate our proposed deterministic continuous VP method on the public and challenging dataset GERMS (Malmir et al., 2015) shown in Figure 4A which is collected in the context of developing robots to interact with toddlers in early childhood education environments. The dataset has 1,365 video tracks of give-and-take trials using 136 different object instances. The object instances are soft toys denoting a wide range of disease-related organisms, microbes and human cell types. Each video track records a robot grasping an object instance to its center of view, rotating the object by 180° with its left or right arm, and then returning it. All video tracks were recorded by a head-mounted camera of the robot at 30 frames/s, as shown in Figure 4B. At the same time, the joint position and object label corresponding to each frame image were also recorded in each track. These joint positions provide an opportunity for verifying different VP methods in one dimensional action space. The dataset authors specified the image subsets of all tracks as train and test set, as shown in Table 1. The evaluation metric used for different VP methods is recognition accuracy that is the average value of the entire test set. The higher the recognition accuracy is, the better the corresponding VP method will be.

**Table 1:** GERMS dataset statistics (mean ± std).

**Images/track**	**Number of tracks**	**Images/track**	**Total number of images**
Train	816	157 ± 12	76,722
Test	549	145 ± 19	51,561

### 4.2. Implementation details

#### 4.2.1. Network architecture

The Tensorflow platform is used to implement the proposed method in this work. In the pre-trained classifier, we transform every image in the GERMS dataset into a 4,096-dimensional feature vector using an existing CNN model VGG-net provided by Malmir et al. (2015). The *softmax* layer has 136 neurons. For the policy network *u*(*s*_*t*_; θ), the dimensions of each layer are 136, 512, 512 and 1. The activation functions of the two hidden layers are both *relu*. The output layer adopts *tanh* activation function, which is multiplied by 512 so as to make the planned relative VP action in [−45°, 45°]. For the two value function networks (*Q*(*s*_*t*_, *a*_*t*_; ω_1_) and *Q*(*s*_*t*_, *a*_*t*_; ω_2_)), they have the same network structure with the dimensions of each layer are 137, 512, 512 and 1. The activation functions of the two hidden layers are also *relu*. The configuration of their corresponding target network is completely consistent with theirs.

#### 4.2.2. Viewpoint transition management

The capacity of the viewpoint transition buffer is 10^6^. ϵ and the exponent λ are set as 0.01 and 0.6 according to the original setting of PER (Schaul et al., 2016). To efficiently sample from distribution (Equation 7), we use a “sum-tree” (Schaul et al., 2016) in which every node is the sum of its children and the leaf nodes are priorities. The sum-tree can be efficiently updated and sampled from.

#### 4.2.3. Training

The reward discount factor γ is 0.95. The minibatch size *N* is 128. The maximum step *T*_*max*_ for recognition is *T*_*max*_ = 12 and the preset probability threshold is 0.99. The Adam optimizer (Kingma and Ba, 2014) is utilized to optimize the policy network and the value function networks. The learning rates are 0.0001, 0.001, and 0.001, respectively. The standard deviations (σ_1_ and σ_2_) of the exploration noise and smoothing regularization are 128 and 32. *c* is 512. The delayed update cycle *d* and soft update τ are 2 and 0.01.

### 4.3. Results and analyzes

#### 4.3.1. Comparison with competing methods

To validate the effectiveness of our proposed deterministic continuous VP method in this experiment, we compare our proposed method with the following baseline and competing methods.

##### 4.3.1.1. Single viewpoint recognition

Single viewpoint recognition only allows the agent to recognize an object from one viewpoint.

##### 4.3.1.2. Blind VP policies

Random policy (Liu et al., 2018a) randomly selects an action from the continuous action space [−45°, 45°] with a uniform probability. Sequential policy (Liu et al., 2018a) moves the agent to the next adjacent viewpoint in the same direction. The reason why these two baseline policies are called blind VP policies is that they do not use the previous observation information for purposeful viewpoint planning. The blind policies may produce worthless viewpoints for recognition.

##### 4.3.1.3. Purposeful discrete VP policy

DQL policy (Malmir et al., 2015; Malmir and Cottrell, 2017) develops an active discrete VP method with deep Q-Learning algorithm, which explores in the discrete action space $\left\{\pm \frac{\pi }{64},\pm \frac{\pi }{32},\pm \frac{\pi }{16},\pm \frac{\pi }{8},\pm \frac{\pi }{4}\right\}$.

##### 4.3.1.4. Purposeful continuous VP policy

TRPO policy (Liu et al., 2018b) utilizes trust region policy optimization (Schulman et al., 2015) to learn a continuous VP policy and adopts extreme learning machine (Huang et al., 2006) to reduce computational complexity. This policy has on-policy characteristic that means the agent can not reuse learned viewpoint transitions for efficient training.

**Figure 5:**
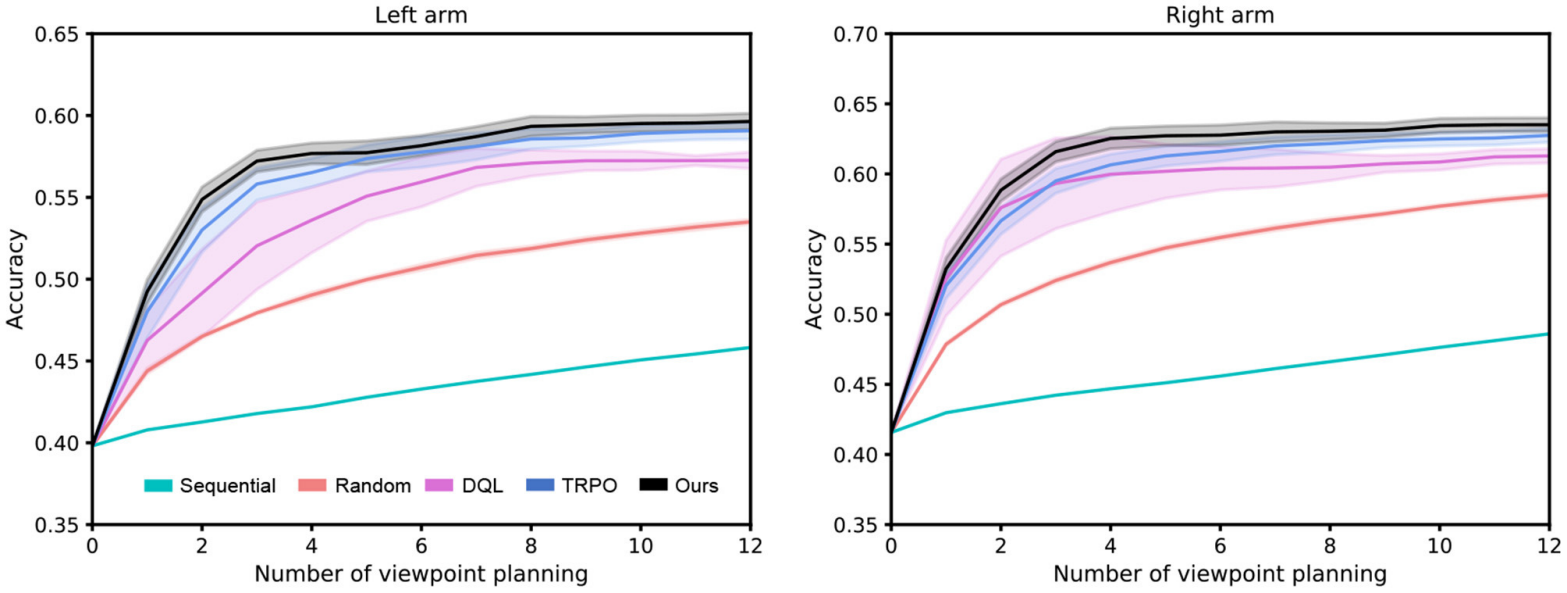
Performance comparison between our presented deterministic continuous VP approach and several competing methods. The shaded region represents the standard deviation of the average evaluation over 10 trials.

**Figure 6:**
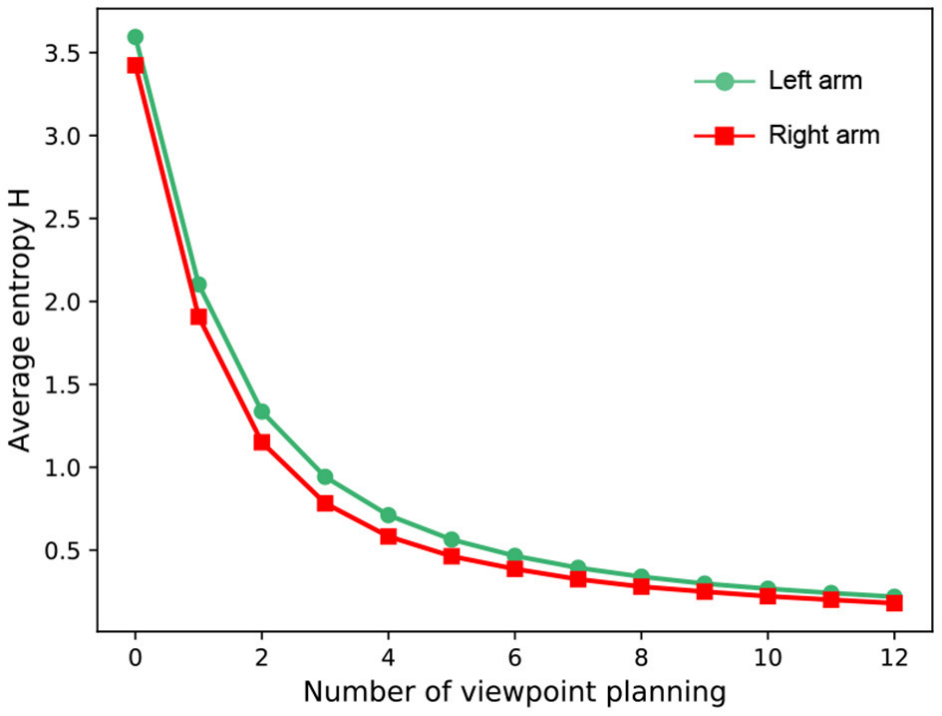
The average entropy over the whole test dataset. The experiment is implemented with our VP model.

**Figure 7:**
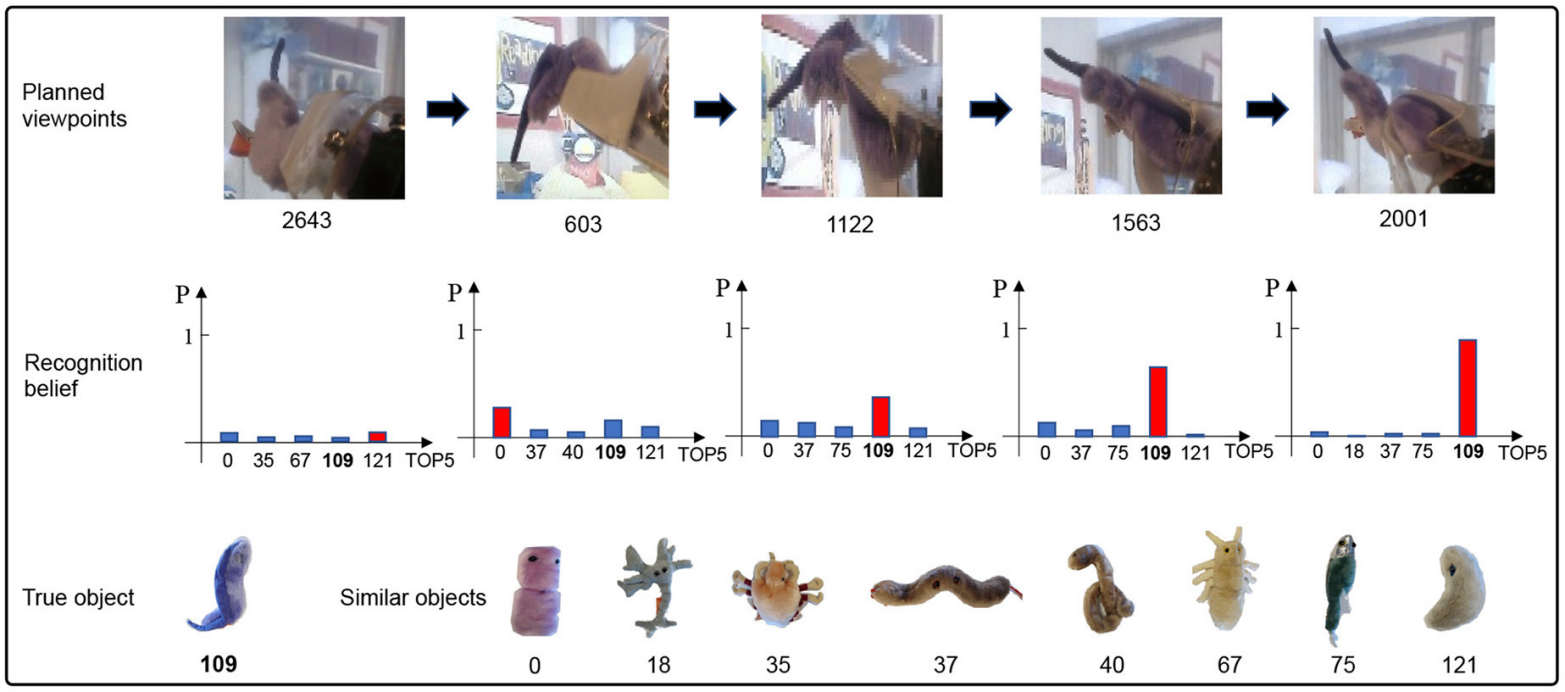
An example of actively identifying an object by our VP method. The recognition belief increases with the increase of the number of viewpoint planning.

Since the main focus of this work is viewpoint planning, we do not investigate the impact of classifiers on recognition performance. Therefore, for a fair comparison, the classifiers in different approaches are the same in the experiment. Figure 5 reports the experimental results of our method against other approaches over 10 random seeds of the policy network initialization. Some observations from Figure 5 are presented as follows: (1) Viewpoint planning can greatly improve recognition performance. The number of VP is 0 that means the agent recognizes the concerned object with a single viewpoint. Obviously, the recognition accuracy of single viewpoint recognition policy is far lower than that of the methods which perform multi viewpoint recognition *via* VP. This is because more object information with difference can be found through VP to reduce recognition uncertainty, thus improving the recognition performance. As shown in Figure 6, the uncertainty of recognition decreases as the number of viewpoints increases. Figure 7 shows the process of actively identifying an object. (2) The performance of the blind VP policies is nowhere near as good as that of the purposeful VP policies. The primary reason is that the purposeful VP policies (i.e., DQL policy, TRPO policy and our policy) can purposefully plan next viewpoints according to the observed information. (3) The continuous VP policies have better performance than the discrete VP policy. That is because the continuous VP policies (i.e., TRPO policy and our policy) directly explore continuous viewpoint space without sampling, so they will not miss some important viewpoints. (4) The performance of our deterministic continuous VP policy exceeds that of TRPO policy. This is mainly because we design a scheme of viewpoint transition management that can reuse the obtained viewpoint transitions to improve the training effect.

#### 4.3.2. Ablation studies

**Figure 8:**
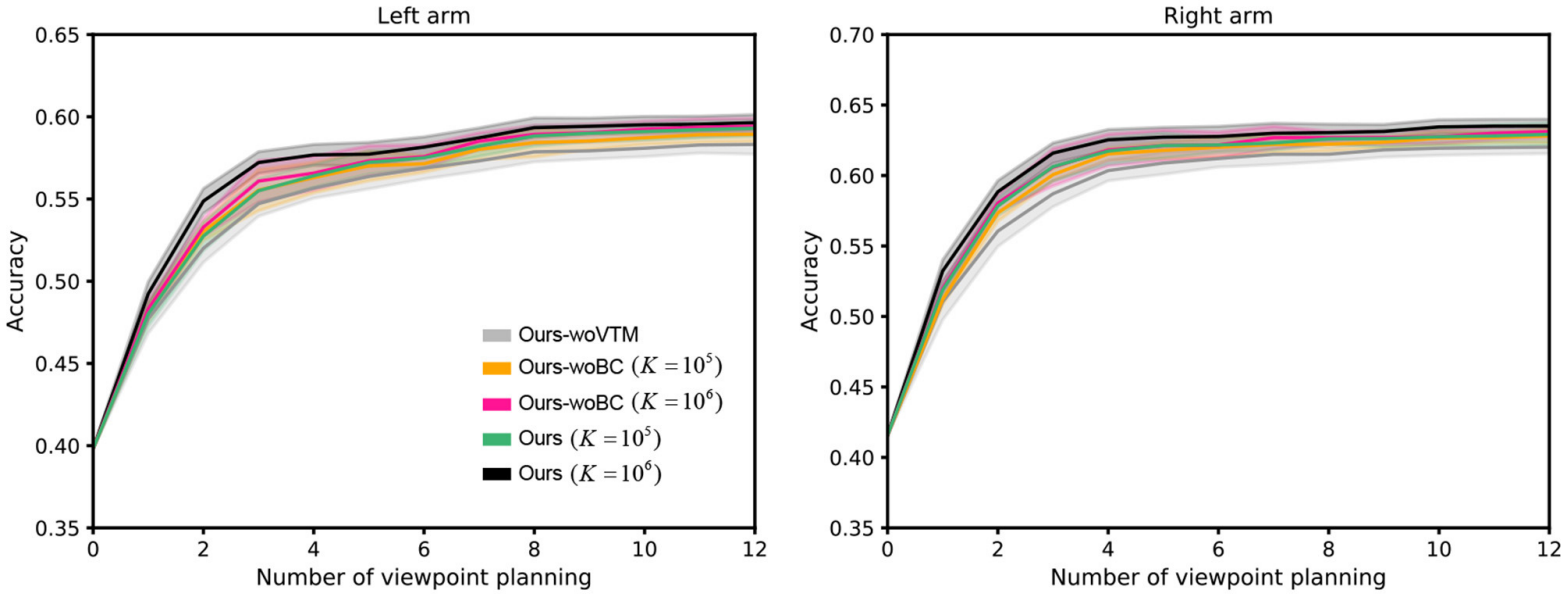
The performance comparison results of ablation experiments. *K* represents the capacity of the viewpoint transition buffer. The shaded region represents the standard deviation of the average evaluation over 10 trials.

To verify the importance of different components in our proposed VP model, we intend to conduct the variant experiments with the ablation of different components, i.e., viewpoint transition management (VTM) and bias correction (BC). Training the model without VTM and BC are respectively denoted as Ours-woVTM and Ours-woBC. From the presented results over 10 random seeds in Figure 8, we can notice that: (1) The performance of Ours-woVTM is the worst. It illustrates that our designed scheme of viewpoint transition management indeed enhances the training effect. (2) The performance of Ours-woBC is inferior to that of Ours, especially when the capacity *K* of the viewpoint transition buffer is large. This is because when the capacity is larger, the distribution of *s*_*t*+1_ in the buffer is closer to its true distribution. In this case, the effect of our bias correction based on importance sampling will be more obvious.

#### 4.3.3. Sampling strategies investigations

**Figure 9:**
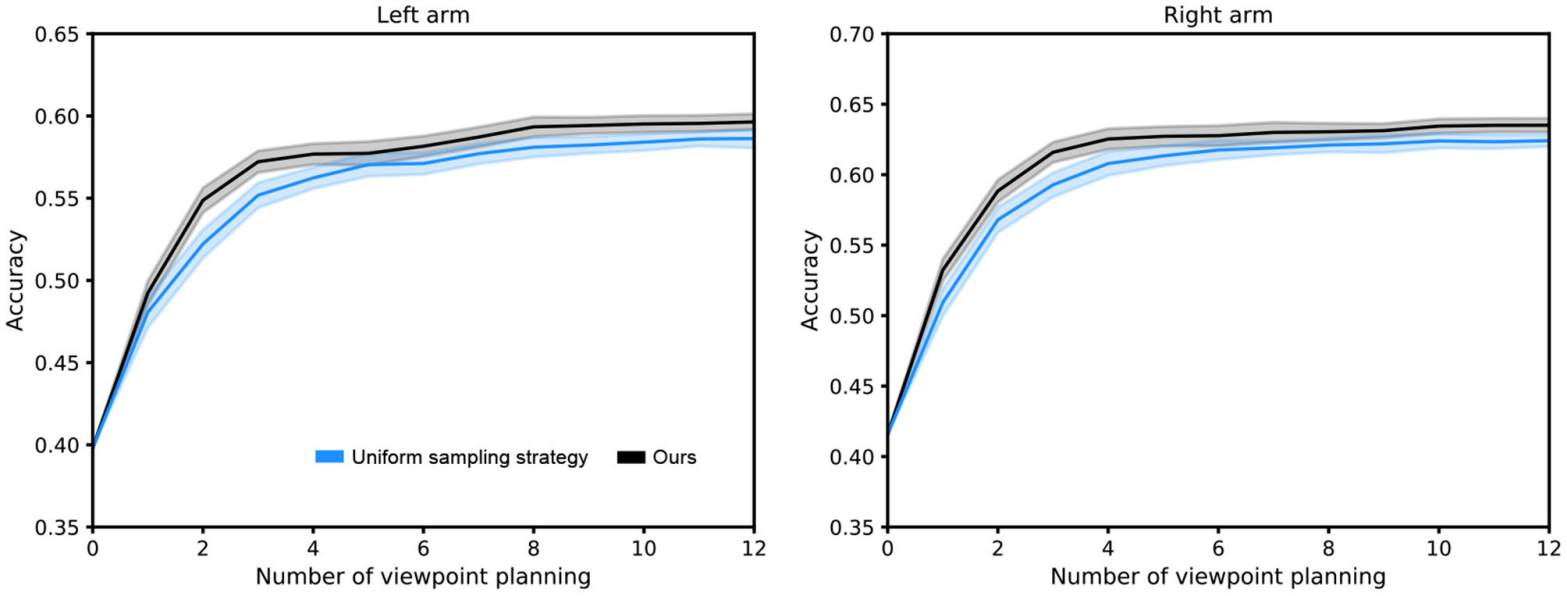
Performance comparison between our sampling strategy and uniform sampling strategy. The capacity of the viewpoint transition buffer is 10^6^. The shaded region represents the standard deviation of the average evaluation over 10 trials.

To verify the superiority of our proposed sampling strategy (i.e., prioritized experience replay based on clipped double Q-learning and bias correction) in the scheme of viewpoint transition management, we conduct comparison experiments with the uniform sampling strategy (Lin, 1992) over 10 random seeds. As shown in Figure 9, we observe that our sampling strategy achieves a better performance, since the importance of each viewpoint transition is ignored by the uniform sampling strategy.

## 5. Conclusions

In this paper, a continuous viewpoint planning method with transition management is proposed for active object recognition based on reinforcement learning. Specifically, we employ deterministic policy gradient theory to build a learning framework of the viewpoint planning policy. We also design a scheme of viewpoint transition management that can store and reuse the obtained transitions. We develop an algorithm based on twin delayed deep deterministic gradient and the designed scheme to train the policy. Experiments on a public dataset demonstrate the effectiveness of our method. In the future, we will integrate the calibrated probabilistic classifiers in AOR research. As stated in Popordanoska et al. (2022), the way the posterior probability distribution is defined in our work assumes that the classifier is properly calibrated, i.e. the *softmax* output represents the correct error rate probabilities. In general, this is not necessarily the case.

## Data availability statement

The original contributions presented in the study are included in the article/supplementary material, further inquiries can be directed to the corresponding author.

## Author contributions

HS and YL: conceptualization. FZ and HS: methodology. HS and YK: software. HS and SF: investigation. FZ: resources and funding acquisition. YL: data curation. HS: writing—original draft. YL and PZ: writing—review and editing. JW and YW: supervision. All authors have read and agreed to the published version of the manuscript.
